# Perspective of Pain Clinicians in Three Global Cities on Local Barriers to Providing Care for Chronic Noncancer Pain Patients

**DOI:** 10.1155/2019/3091309

**Published:** 2019-02-03

**Authors:** S. Fatima Lakha, Peri Ballantyne, Hanan Badr, Mubina Agboatwala, Angela Mailis, Peter Pennefather

**Affiliations:** ^1^Institute of Medical Science, University of Toronto, Toronto, Canada; ^2^Department of Sociology, Trent University, Faculty of Medicine, Peterborough, Canada; ^3^Department of Community Medicine, Kuwait University, Kuwait, Kuwait; ^4^HOPE, Karachi, Pakistan; ^5^Pain & Wellness Centre, Vaughan, Canada; ^6^Leslie Dan Faculty of Pharmacy, University of Toronto, Toronto, Canada

## Abstract

An increasing proportion of the global chronic pain population is managed through services delivered by specialized pain clinics in global cities. This paper describes the results of a survey of pain clinic leaders in three global cities on barriers influencing chronic noncancer pain (CNCP) management provided by those clinics. It demonstrates a pragmatic qualitative approach for characterizing how the global city location of the clinic influences those results. A cross-sectional prospective survey design was used, and data were analyzed using quantitative and qualitative content analysis. Key informants were pain clinicians (*n* = 4 women and 8 men) responsible for outputs of specialized pain clinics in academic hospital settings in three global cities: Toronto, Kuwait, and Karachi. Krippendorff's thematic clustering technique was used to identify the repetitive themes in the data. All but one of the key informants had their primary pain training from Europe or North America. In Kuwait and Karachi, pain specialists were anesthesiologists and provided CNCP management services independently. In Toronto, pain clinic leaders were part of some form of the multidisciplinary team. Using the results of a question that asked informants to list their top three barriers, ten themes were identified. These themes were artificially organized in three thematic domains: infrastructure, clinical services, and education. In parallel, 31 predefined barriers identified from the literature were scored. The results showed variation in perception of barriers that not only depended on the clinic location but also demonstrated shared experiences across thematic domains. This study demonstrates a simple methodology for informing global and local efforts to improve access to and implementation of CNCP services globally.

## 1. Introduction

Chronic noncancer pain (CNCP) has become a serious public health issue affecting more than 70 million people, globally [1]. It can have a multitude of causes and produces a wide variety of disabilities [2]. Several studies have shown substantial variability in the way clinicians approach and treat CNCP [3–5]. However, pain management practice guidelines have been developed and disseminated [2, 6–8] that recommend meeting pain-related health-care needs through accessing specialized pain clinics. Although a substantial proportion of the global CNCP population has pain-related health-care needs met through accessing specialized pain clinics [9], little or no information exists on the experience and perception of clinicians in those clinics with respect to the factors that impede their specialty practices.

Despite the fact that research and clinical studies conducted in many countries on all aspects of pain services have been published, factors affecting the output or functioning of the pain management services remain poorly described and understood [10]. Furthermore, published studies have not explored how barriers can emerge from the interaction between the specialized clinical training of the clinicians, often received elsewhere, and the local institutional settings where they practice [11–13]. Pain specialists working in clinics providing CNCP services are uniquely poised to serve as key informants on these issues. We have implemented a pragmatic qualitative descriptive approach to gain insight into work demands in clinical areas and levels of accountability surrounding pain management. This report is part of a larger study that aimed to compare and characterize the experience and perspectives of pain specialists practicing in the three cities. This report focuses on barriers that they faced in delivering clinical services for people living with CNCP.

Many global cities [14] have access to highly qualified medical specialists, including pain specialists who practice in well-equipped academic medical centers. Practitioners within such centers should have comparable opportunities for improving the well-being of people seeking relief from CNCP symptoms. This study is part of research project exploring opportunities and challenges for improving the availability of CNCP services globally, using a global cities lens with an initial focus on cities in the region served by the Eastern Mediterranean Region Office of the World Health Organization (EMRO-WHO) [15]. The wide diversity of wealth and social status amongst inhabitants of those cities is likely to impact on outcomes of chronic pain management processes. Studying that impact should inform how pain management practices can be globalized. Accordingly, we set out to demonstrate the feasibility of comparing and characterizing the experience and perspectives of pain specialists practicing in global cities regarding barriers that they faced in delivering clinical services for people living with CNCP.

Our initial focus was on two global cities in this region, Karachi and Kuwait City, and Toronto, a Canadian global city with a large population originating from the region, and is the home of the group leading this research. Each of these global cities is investing in developing and maintaining health-care systems in which global best practices are accessible. As these global best practices are not regulated at the global level, they will necessarily be adapted, in ways influenced by local contexts and constraints. We seek to uncover and map similarities and differences in that adaptation process. The terms *pain clinic leaders*, *pain specialist,* and *pain clinicians* are used interchangeably to characterize clinicians practising in a self-identified specialized pain clinic affiliated with an academic teaching hospital, independently of any formal certification.

## 2. Methodology

### 2.1. Study Design and Setting

A cross-sectional prospective semistructured questionnaire was used to anchor a qualitative analysis of barriers to services for CNCP experienced by pain specialists practicing in academic hospitals settings hosting a specialized pain clinic staffed with health-care professionals who have completed certified fellowships in pain management. Appropriate local ethics approval was gained from all three global cities, and all participants signed consent forms.

In the first section of the questionnaire, the participants were asked about their training and if their clinic offered services for the treatment of (1) acute pain, (2) chronic noncancer pain, and/or (3) cancer pain and/or (4) paediatric pain. The criterion for inclusion of a pain specialist was his or her involvement in the delivery of CNCP management services 6 months prior to completion of the questionnaire. Key informants who provide care exclusively in paediatric or cancer pain departments were excluded. Based on the above criteria, semistructured, guided interviews were carried out by the lead author (SFL) with a convenience sample of clinics in Kuwait City (*n* = 4), Karachi (*n* = 4), and Toronto (*n* = 4). Participants were asked to reflect only on their experiences in public clinic settings. In this study, participants are recruited based on preselected criteria determined by the research question. A convenience sample of 3 global cities, where we had contacts (Toronto, Karachi, and Kuwait), could provide a sufficient number of key informants for the study. It is generally accepted that with homogeneous groups, structured interviews, and a concrete research question, saturation generally occurs with around 12 participants [16].

### 2.2. Study Questionnaire

The study questionnaire was designed to gather information in terms of structural elements, clinical care processes, and barriers to delivery of services. The questionnaire was pilot-tested in one hospital in Toronto. The questionnaire sections were rooted in questions found in well-established research instruments [17, 18]. It was delivered in English and had three parts: Part I asked questions about the background of the institutions and key informants; Part II covered the organizational structure and clinical activities associated with the pain services.

The results reported and analyzed in this article dealt with a portion of Part III composed of two sections: section (a) provided an opportunity to list their top three barriers (this section sought single-phrased responses to the open-ended questions concerning key informants' perceived three top barriers), and section (b) provided a list of barriers related to the infrastructure, clinical services, education, and training developed from a prior attempt to systematically review the literature concerning local differences in CNCP practice across different global cities [14]; each listed barrier could be scored using a Likert scale, starting from 0 = not a barrier to 4 = extreme barrier (see Appendix 1).

### 2.3. Data Collection

The first part of the data were collected using a self-administered questionnaire about key informant demographics and clinical practices filled in by the key informant during the first part of the interview. Subsequently, individual semistructured face-to-face interviews were conducted. This was carried out in a consulting location at the pain clinic and audio recorded. The key informants were first asked to describe principal barriers limiting satisfactory operation of pain programs in general. The opening question of this part of the interview was “state three principal barriers you face while delivering pain management services at your clinics.” Subsequent to their identification of the three barriers, a comprehensive list of perceived barriers, extracted from a review of the literature, was given to the key informants for their rating.

### 2.4. Data Analysis

**Figure 1:**
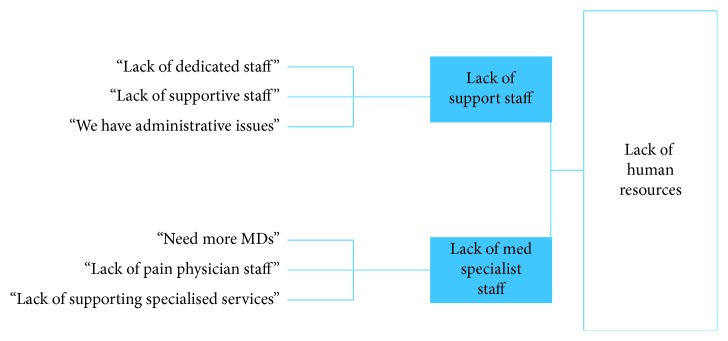
Example of dendrogram.

Descriptive statistics were used to describe the general characteristics of the institutions and key informants demographics and training. Comparisons of barriers across the sites were done using contingency tables. Interviews were transcribed from audio recordings, entered as text, and coded using QSR NVivo Software [19]. Text data describing informants' top three barriers were subjected to Krippendorff's method to identify repetitive themes in the content [20]. Led by SFL, data were grouped according to Krippendorff's analytical technique of clustering to identify phrases and sentences that shared some characteristics. As an example, statements such as “lack of support staff,” “need more MDs,” and “lack of specialized services” were categorized as lack of human resources theme. Dendrograms, or tree-like diagrams, were created to illustrate how clusters were grouped into themes and are presented in Figure 1. Two coauthors (PP and AMG) reviewed the text data and content in order to validate the clusters and themes. The analysis was finalized by identifying several themes that emerged from the specific description of barriers. Regarding trustworthiness of the themes, credibility was established through a validation process, after having analyzed a series of interviews. After reliably retrieving the most salient themes from each of the three sites in 12 interviews, we established what we call among and within-group data saturation. To analyze responses to the list of 31 barriers to treating CNCP patients, the magnitude assigned to each perceived barrier was computed by aggregating responses to the Likert scale. For qualitative comparison, mean scores were pragmatically aggregated as follows: mild barriers (0–2), moderate barriers (>2<3), and severe barriers (3-4).

## 3. Results

### 3.1. General Characteristics

All pain clinics were located in large university-affiliated hospitals, in core urban areas of Toronto, Kuwait, and Karachi. In Kuwait and Karachi, informants/pain specialists provided the services in a solo practice, while in Toronto, informants/clinicians were part of some form of a multidisciplinary team (pain physician, a nurse, and a psychologist/or physical therapist). All informants were responsible for pain clinics that offered services for the management of chronic pain, but the specifics of those services varied considerably across sites.

Information was gathered from 12 key informants (4 women and 8 men). Key informants' age ranged from 36–64 years (Toronto), 36–55 years (Kuwait), and 46–55 years (Karachi). All key informants graduated from English universities and had (with one exception) received their primary pain management training at medical schools in Europe or North America. All key informants from Kuwait and Karachi were anesthesiologists. In contrast, each of the key informants from Toronto had different specializations (i.e., anesthesiology, family medicine, physical medicine, and rehabilitation). In Toronto, the time in pain practice for key informants ranged from 15 to 25 years, while in Kuwait and Karachi, the average time in pain practices ranged from 5–15 years.

### 3.2. Principal Barriers for Pain Management in the Pain Clinics

Ten general themes were identified that accommodate all of the key barriers reported at all sites for managing CNCP. The themes are artificially organized into three domains: infrastructure, clinical services, and education. They correspond with the larger domains of structure, process, and output for which evidence exists in the literature [15, 21]. The themes are summarized from single-phrased responses about the three principal barriers and outlined in Table 1. Table 1 compares and contrasts the themes of principal barriers for the management of CNCP among the three global cities.

#### 3.2.1. Infrastructure

The term “infrastructure” is understood to refer to the structural and operational framework of an institution [22]. It is used to cover three themes (#1, #2, and #3) recognized in informant responses: scarcities in general resources, lack of human resources or personnel in the pain clinic, and obstacles emerging from structures of the hospital system in which the clinic functioned.

*Theme one: lack of access to general resources by the pain clinic:* Key informants in each city identified limitations in access to general resources as an important barrier for delivery of CNCP management services. They reported dearth of supplies, inadequate funding, and lack of infrastructure as barriers. Structural issues were noted such as “lack of dedicated space” or “space for pain clinic.” In addition, informants from Kuwait and Karachi also cited the limited availability of equipment and supply services. A key informant from Toronto indicated that his/her center lacked a model of multidisciplinary care and offered that it was “desperately needed.” The key informant stated that delivery of CNCP management services would be improved if hospitals implemented a standardized multidisciplinary service delivery model.

*Theme two: lack of human resources in the pain clinic:* The staffing shortage in the pain clinic related to two components: lack of support staff and lack of access to medical/other specialists. Key informants from across the study sites mentioned the lack of support staff (such as administrative and secretarial) and dedicated staff support (such as nurses) for the pain clinic operations. The pain clinicians seemed to feel isolated and unsupported. The participants further reported a lack of access to other supporting specialized services (psychologist, psychiatrists, and/or physiotherapist) in the pain clinic or hospital. A key informant from Toronto stressed the need for greater access to dedicated services within the pain clinics and emphasizing the need for psychological services and mental health support. Two key informants, one from Karachi and one from Toronto, each emphasized the need for more pain management specialists in the clinics.

*Theme three: obstacles in hospital system:* This is another theme mentioned by one informant in each city related to obstacles caused by management and operations of the hospitals hosting the pain clinic. Key informants mentioned the unwillingness of hospital administrators to provide or expand support for pain clinic operations.

A key informant from Toronto reported that hospital policies prevented delivery of simple pain-reducing interventional procedures such as injections in the space provided for consultation. The comment that reflected this is as follows:

“… . .Pain specialist cannot do simple procedure (injections) in the pain clinic.”

#### 3.2.2. Clinical Services

The clinical services domain consists of three themes (#4, #5, and #6) that cluster around the relationship among the providers and reflect upon the regular practices of pain specialists managing patients with CNCP, and the impact of this work on them as individuals and clinicians.

*Theme four: lack of communication/collaboration by providers:* Toronto's key informants identified lack of communication among interprofessional teams as a barrier, while this was not the case for key informants from Kuwait and Karachi as they work individually. A key informant from Toronto emphasized the need for better collaboration and cooperation across the city among pain management physicians and programs.

*Theme five: patient issues:* Patient issues in clinical services fell into two areas: cultural barriers reflecting limitations arising from cultural factors that influenced how clinical practices were implemented (*e.g.*, “male physicians cannot see female patients etc.”) and patient expectations concerning what they believed the clinic should be doing for them (e.g., “taking away their pain”). A key informant from Toronto reported difficulties in communicating with patients due to cultural barriers. Furthermore, key informants from Kuwait perceived patients as having unrealistic expectations regarding outcomes of pain management. “…High patient's expectation for care.”

*Theme six: system barriers:* Findings in this theme include excessive demand for services, lack of financial support for providers, and patients' lack of financial means to pay for medication, procedure, and lack of access to opioids. A key informant from Kuwait mentioned that they receive overwhelming referrals for pain management services from all over Kuwait and other parts of the Gulf. A key informant from Karachi reported, “… .the salary scale for providers is so horrible, that is why doctors do not come to this field.” A Karachi key informant stressed lack of funds for poor patients to buy drugs or access to pain management interventions, and even if funds were available, there is only limited access to strong opioids and morphine. Lack of access to opiates was also a concern to key informants in Kuwait.

#### 3.2.3. Education

The education domain encompassed four themes (#7, #8, #9, and #10) that cluster around the professional development of the pain management specialists, actual training of those who claim to manage CNCP, CNCP knowledge and awareness among general physicians, and CNCP knowledge among the general population.

*Theme seven: no systematic pain management education:* Only key informants from Toronto referred to a dearth of systematic educational and training programs around CNCP best practices for general health-care workers and pain specialists.

*Theme eight: lack of actual pain management knowledge:* Participants in all three cities mentioned that there is a disparity in the training and level of knowledge of pain professionals working in the pain clinics. This is exemplified by an assertion from a key informant that “pain practitioners are not truly trained, but they claim to be.”

*Theme nine: lack of pain management knowledge among general/primary care physicians:* Five key informants from Karachi and Kuwait identified inadequate CNCP knowledge and training among primary care physicians and family physicians. Specifically, they referred to a lack of awareness about CNCP management methods, pain clinics, and other resources among general physicians.

*Theme ten: lack of knowledge CNCP management opportunities by the general public:* A key informant from Karachi highlighted the lack of general education among poor patients concerning health-care concepts. This lack impacts the patients' responsibility for participating in the pain management process. Key informants from Karachi and Kuwait also mentioned the lack of awareness about CNCP management among the general population.

“… Lack of awareness about chronic pain management among general population.”

“… .Society is immature for pain specialty.”

### 3.3. Key Informants' Perception of Barriers for Managing CNCP

The items in Table 2 (a, b, and c) are coded according to a grey scale determined by mean values of Likert scale responses to thirty-one barriers listed in Part III of the questionnaire. “Lack of psychological and social support services” (under the domain of infrastructure) and “Coordination of care” (under the domain of clinical services) were perceived as severe barriers by all the respondents in all global cities. The barriers that scored mild to moderate in all global cities were social, cultural, regulatory, and access barriers.

Barriers perceived as severe in Karachi and Kuwait but not Toronto were “excessive-regulation of access to opioids,” “patient adherence to treatment,” “lack of awareness of the value of referrals to pain clinics” (under the domain of clinical services), “lack of awareness about pain management among patients,” and “lack of staff knowledge and knowledge about pain resources among general physicians” (under the domain of education). Barriers perceived as severe in Kuwait and Toronto but not Karachi were “lack of time” and “access to resources” (under the domain of infrastructure). Barriers shared by Toronto and Karachi but not Kuwait included the cost of medications, training, and education of staff and travel time to reach the clinic (under the domain of infrastructure and education). Perceived barriers for CNCP management were rated high by Karachi key informants while key informants from Toronto rated them the lowest in all three domains.

## 4. Discussion

To the best of our knowledge, this is the first comparative description of specialized pain clinics providing CNCP management services from academic hospitals located in different global cities. This study illustrates a simple methodology for revealing an explanatory picture of globally distributed pain specialists' experiences and perceptions of barriers about CNCP management. Despite differences in the social, economic, and cultural characteristics of the EMRO countries (Kuwait and Pakistan) and Canada, many common elements were shared regarding the experience and perception of barriers. This is perhaps not surprising as, but one key informant was trained in Europe or North America, and all received similar specialist training. Through the application of a pragmatically structured qualitative description method, we were able to identify and elaborate on three distinct domains relating to pain management practices within academic hospitals: 1) *infrastructure,* 2) *clinical services,* and 3) *education*. The study also demonstrated some interesting but understandable differences in prioritizing barriers to be overcome in each of the three global cities. Since this study was conducted in the context of principal barriers about pain management in their respective pain clinics and cities, any key informant may have perceived a barrier not reported by others in that global city.

Across the three domains, multiple barriers were identified; many of those barriers were experienced across all three global cities. Infrastructure issues included lack of access to resources and collaboration with allied health professionals. Well-developed comprehensive treatment plans were identified as being impeded by hospitals system barriers. Clinical services issues focused on the interpersonal aspects of provider-patient interaction to pain care. The multiple specific themes within this domain can highlight targets for improvement in local settings. Education issues related to lack of awareness among the general public and other health-care providers are associated with CNCP care. Qualitative analysis indicated an urgent need for more education development initiatives for the clinicians and staff. It is also evident that overall Karachi scores were the highest in terms of perceived barriers and Toronto were the lowest. The themes and survey highlighted the complexity of managing CNCP, and difficulties routinely faced by clinicians are in line with the results reported by Lalonde et al. [23]. Additionally, the study provides a picture of the challenges and opportunities for improving clinical care for CNCP patients and a methodology for examining the globalization of that care.

### 4.1. Suggestions for Overcoming the Barriers Themes Related to the Infrastructure Domain

Our results illustrate how a systemic lack of awareness of widely recognized programmatic standards for delivering specialized CNCP services forces each clinic leader to navigate his or her own path alone. Many issues related to administration and human resources cited in this study might be improved through better integration of a health-care team model supported by the institutional host. Pain clinic leaders perceived a need to train more pain medicine specialists, as the supply of pain specialists appears to be declining [24]. The use of nurses in providing support for patients with chronic pain has been shown to improve patient satisfaction and pain scores [25–27]. Timely referrals for consultations with allied health-care professionals are known to reduce the use of medication, improve patient's self-management, and the outcomes of health-care consultations [28]. Other collaborative and interdisciplinary approaches beyond pain management may help patients with complex psychosocial and behavioral issues, as chronic pain is prevalent in two-thirds of patients with major depressive illness [29, 30].

In the last decades, the multidisciplinary approach to pain management has become popular, but the emergence of subspecialization in pain management in many clinical specializations has also led to variations and fragmentation of care depending on which specialist takes the lead establishing the pain clinic [31]. Fortunately, health-care authorities of several jurisdictions have recognized the need for establishing a uniform standard of training and certification for pain specialists' across discipline emphasizing interprofessional collaboration [32].

### 4.2. Suggestions for Overcoming the Barriers Themes Related to the Clinical Services Domain

In the Clinical Services theme, perceived barriers included difficulties in communicating with patients and addressing differing expectations between patients and providers regarding the effects of pain care; this challenge has been discussed by others [33, 34]. To overcome these barriers, all pain clinicians from the three global cities recognized a need for better training in handling challenging encounters, at least learning how to integrate behavioral management practices provided by allied health professionals.

### 4.3. Suggestions for Overcoming the Barriers Themes Related to the Education Domain

To address educational barriers with regard to pain management for both health providers and general population, certain strategies should be employed. University-level interdisciplinary continuing education programs [23, 35, 36] should be made available to all pain management clinicians specialists and allied health professionals seeking to become practice leaders in this area. Certification and standardization of pain specialist programs across specializations and disciplines should be prioritized by health-care planners and policy makers globally. This study demonstrates a methodology for beginning to recognize the scope of the challenge.

### 4.4. Limitations of the Study

While the study offers unique data from academic pain clinics in global cities, our convenience sample of only three global cities in this proof of concept suggests that generalizations to other global cities or other settings should be made with caution. Indeed, the approach is designed to enable structured comparisons that can bring forth the uniqueness of each context, while identifying cross-cutting themes. Any interpretation and use of results should take this into consideration. The sample is deliberately small, nonrandom, and limited to clinical leaders at academically affiliated specialized pain clinics in global cities. As in all self-report research, the findings need to be supported by further detailed observational studies involving other global cities from different regions.

## 5. Conclusion

Chronic pain management gets limited attention in medical training, in research, and in administration of health-care institutions despite recognition of its widespread prevalence and large global burden. Findings from our study provide a new lens on mapping barriers to improving delivery of clinical care for CNCP conditions experienced by people seeking help from specialized pain clinics in global cities. Our approach to mapping barriers is easy to apply and demonstrates how shared concerns informed by the realization of local constraints could guide the development of international best practice guidelines that can be adapted to local constraints.

## Figures and Tables

**Table 1:** Principal barriers in pain programs.

Domain	Themes	Toronto	Kuwait	Karachi
Infrastructure	(1) Lack of general resources	x	x	x
	(2) Lack of human resources	x	x	x
	(3) Obstacles in hospital systems	x	x	x

Clinical services	(4) System barriers	—	x	x
	(5) Patients issues	x	x	
	(6) Lack of communication/collaboration among providers	x	—	—

Education	(7) Shortage of systematic pain management education program	x	—	—
	(8) Lack of pain management knowledge among pain clinic staff	x	x	x
	(9) Lack of pain management knowledge by general physicians	—	x	x
	(10) Lack of education of patient population	—	x	x

x: at least one key informant from the city indicated by the column heading reported a barrier that could be assigned to the barriers theme row.

**Table 2:** Perception of Barrier for Managing CNCP.

	Toronto	Kuwait	Karachi
*2(a) Infrastructure*			
(i) Psychological and social support services	3.25	3.75	3.75
(ii) Lack of access to interventions (blocks, spinal stimulators, etc.)	2.25	2.25	2.75
(iii) Lack of time and resources to address noncancer pain	3	3.25	2.75
(iv) Access to assessment of patients with CNCP	1.5	1.5	2.75
(v) Clinic too far or inconvenient for patients' to travel to	3	1.75	3.25
(vi) High cost of medications and treatments	3	0.75	4
(vii) Lack of access to wide range neuropathic adjuvant medications (e.g., gabapentin, pregabalin, duloxetine)	2.25	1.5	1.75
(viii) Access to wide range of opioids	1	3	3
(ix) Regulation of opioids by Narcotics Bureau, Dept. of Health	0	3.25	3.25
(x) Excessive regulation of opioids in pharmacy	0	3	3.5
(xi) Waiting list to see physicians/specialists	3	2.5	2.75
(xii) Regulatory barriers to effective pain management	1.5	2.75	2.75

*2(b) Clinical Services/Practices*			
(i) Coordination of care, particularly acute to chronic transition	3.25	3.5	3
(ii) Patient and family fear that reporting pain will exclude a patient from clinical trials or treatment	1	1.5	2
(iii) Patients' reluctance to take opioids	2	2.25	3
(iv) Legal and regulatory sanctions for opioid use	0.5	2	3.5
(v) Inadequate reimbursement for providers	1.75	0.5	2.25
(vi) Patient and family failure to mention pain to providers	1	1	2.5
(vii) Religion (e.g., male physicians cannot see female patients, etc.)	1	0.5	2.5
(viii) Cultural barriers to accepting taking pain medications	1	2	2.5
(ix) Cultural barriers (e.g., male patients do not complain as they think pain is a sign of weakness)	1	1.25	2.25
(x) Physicians' reluctance to prescribe opioids	0.5	3.75	3
(xi) Patient's fear drugs will lose their effectiveness	2.25	3.25	3
(xii) Patient adherence to treatment regimens	2.75	3.25	3
(xiii) Lack of public awareness about the presence of pain clinic	2.25	3.5	3.75
(xiv) Cognitive impairment hindering assessment	1.75	2	2.5

*2(c) Education*			
(i) Inadequate CNCP management training and education of staff	3	1	3
(ii) A priority on curing noncancer pain over managing	3.75	2.25	3
(iii) Knowledge about available resources	2.5	3.25	3
(iv) Awareness of other physicians about pain clinic benefits for referral purposes	1.75	3.5	3.75
(v) Inadequate staff knowledge of pain management	2	3.25	3.25

Likert scale compression: 0–2 (mild); >2<3 (moderate); 3–4 (severe); values indicate mean score with *N* = 4 from each studied.

## Data Availability

The raw qualitative data used to support the findings of this study are restricted by the Ethics Boards of University of Toronto, Kuwait University, and Hope Institution in order to protect the key informants and institutions identities.

## Appendix

### Questionnaire

Services for management of chronic noncancer pain (CNCP) in global cities (Part III)—BARRIERS.

**Table:**

(D) BARRIERS

What are the 3 principal barriers for you in the pain program?
(1)
(2)
(3)
Comments:

What barriers/difficulties you have in managing CNCP? Rate the intensity of the barriers (0 being not a barrier and ++++ as an extreme barrier)—MARK ALL THOSE THAT APPLY.

**Table:**

	0	++	+++	++++

*Infrastructure*				
Psychological and social support services				
Lack of access to interventions (blocks, spinal stimulators etc.)				
Lack of time and resources to address noncancer pain				
Access to assessment of patients with chronic noncancer pain				
Clinic too far or inconvenient for patient to travel to				
High cost of medications and treatments				
Lack of access to wide range neuropathic adjuvant medications (e.g., Gabapentin, pregabalin, duloxetine)				
Access to wide range of opioids				
Excessive regulation of opioids in Narcotics Bureau, Department of Health				
Excessive regulation of opioids in pharmacy				
Waiting list to see physicians/specialists				
Regulatory barriers to effective pain management				

*Clinical Services/Practices*				
Coordination of care, particularly during transition from acute to chronic				
Patient and family fear that reporting pain will exclude patient from clinical trials or treatment				
Patients' reluctance to take opioids				
Legal and regulatory sanctions for opioid use				
Inadequate reimbursement for providers				
Patient and family failure to mention pain to providers				
Religious barrier (e.g., Male physicians cannot see female patients etc.)				
Cultural barrier for pain medications				
Cultural barriers (e.g., Male patients do not complain as they think pain is sign of weakness)				
Religious barriers (e.g., Male physicians cannot see female patients etc.)				
Physicians' reluctance to prescribe opioids				
Patient's fear drugs will lose their effectiveness				
Patient adherence to treatment regimens				
Lack of awareness among patients and families about presence of pain clinic				
Cognitive impairment hindering assessment				

*Education*				
Inadequate noncancer pain management training and education				
A priority on curing noncancer pain over managing				
Awareness of other physicians about pain clinic benefits for referral purposes				
Knowledge about available resources				
Inadequate staff knowledge of pain management				
